# 4-(3-Methyl-5-phenyl-1*H*-pyrazol-1-yl)benzene­sulfonamide

**DOI:** 10.1107/S1600536811032855

**Published:** 2011-08-27

**Authors:** Abdullah M. Asiri, Hassan M. Faidallah, Abdulrahman O. Al-Youbi, Salem A. Basaif, Seik Weng Ng

**Affiliations:** a Chemistry Department, Faculty of Science, King Abdulaziz University, PO Box 80203 Jeddah, Saudi Arabia; b Center of Excellence for Advanced Materials Research, King Abdulaziz University, PO Box 80203 Jeddah, Saudi Arabia; c Department of Chemistry, University of Malaya, 50603 Kuala Lumpur, Malaysia

## Abstract

With respect to the planar five-membered ring of the title compound, C_16_H_15_N_3_O_2_S, the phenyl ring is aligned at 47.0 (1)° and the phenyl­ene ring at 37.6 (1)°. The amino group has the N atom in a pyramidal geometry; the group is a hydrogen-bond donor to the sulfonyl O atom of one mol­ecule and to the pyrazole N atom of another mol­ecule, resulting in the formation of a layer parallel to the *bc* plane.

## Related literature

For the synthesis, see: Gosselin *et al.* (2006 ▶); Organ & Mayer (2003 ▶).
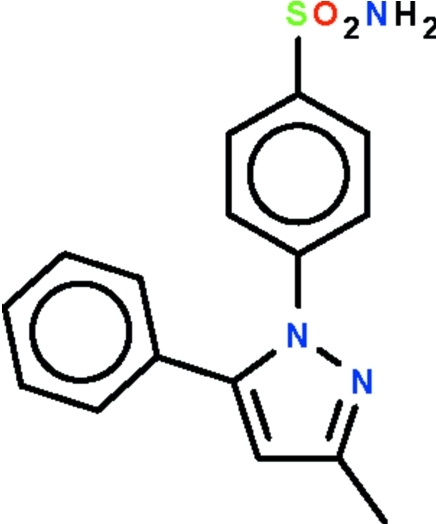


## Experimental

### 

#### Crystal data

C_16_H_15_N_3_O_2_S
*M* *_r_* = 313.37
Monoclinic, 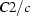

*a* = 28.2545 (8) Å
*b* = 11.9135 (4) Å
*c* = 9.3739 (3) Å
β = 91.016 (3)°
*V* = 3154.85 (17) Å^3^
*Z* = 8
Cu *K*α radiation
μ = 1.91 mm^−1^
*T* = 100 K
0.30 × 0.03 × 0.03 mm

#### Data collection

Agilent SuperNova Dual diffractometer with an Atlas detector
Absorption correction: multi-scan (*CrysAlis PRO*; Agilent, 2010 ▶) *T*_min_ = 0.598, *T*_max_ = 0.945
6579 measured reflections
3137 independent reflections
2689 reflections with *I* > 2σ(*I*)
*R*_int_ = 0.035

#### Refinement

*R*[*F* ^2^ > 2σ(*F* ^2^)] = 0.041
*wR*(*F* ^2^) = 0.115
*S* = 1.03
3137 reflections
208 parameters
H atoms treated by a mixture of independent and constrained refinement
Δρ_max_ = 0.33 e Å^−3^
Δρ_min_ = −0.51 e Å^−3^

### 

Data collection: *CrysAlis PRO* (Agilent, 2010 ▶); cell refinement: *CrysAlis PRO*; data reduction: *CrysAlis PRO*; program(s) used to solve structure: *SHELXS97* (Sheldrick, 2008 ▶); program(s) used to refine structure: *SHELXL97* (Sheldrick, 2008 ▶); molecular graphics: *X-SEED* (Barbour, 2001 ▶); software used to prepare material for publication: *publCIF* (Westrip, 2010 ▶).

## Supplementary Material

Crystal structure: contains datablock(s) global, I. DOI: 10.1107/S1600536811032855/bt5609sup1.cif

Structure factors: contains datablock(s) I. DOI: 10.1107/S1600536811032855/bt5609Isup2.hkl

Supplementary material file. DOI: 10.1107/S1600536811032855/bt5609Isup3.cml

Additional supplementary materials:  crystallographic information; 3D view; checkCIF report

## Figures and Tables

**Table 1:** Hydrogen-bond geometry (Å, °)

*D*—H⋯*A*	*D*—H	H⋯*A*	*D*⋯*A*	*D*—H⋯*A*
N1—H1⋯N3^i^	0.92 (2)	1.98 (2)	2.878 (2)	164 (2)
N1—H2⋯O1^ii^	0.86 (2)	2.07 (2)	2.930 (2)	177 (2)

Symmetry codes: (i) 
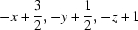
; (ii) 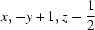.

**Fig. 1.**
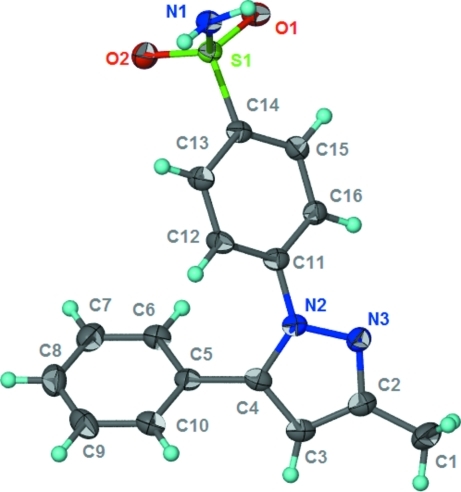
Anisotropic displacement ellipsoid plot (Barbour, 2001) of C16H15N3O2S at the 70% probability level; hydrogen atoms are drawn as spheres of arbitrary radius.

## supplementary crystallographic information

### Comment

We are examining the medicinal properties of phenylpyrazolones of which the 4-benzenesulfamide derivative (Scheme I) is expected to show enhanced activity. As the inhibitory activity against cyclooxygenase-1 and cyclooxygenase-2 of the title compound (Scheme I) has been claimed in a number of patents, other researchers have attempted its synthesis in order to increase yield (Gosselin *et al.*, 2006; Organ & Mayer, 2003). With respect to the planar five-membered ring, the phenyl ring is aligned at 47.0 (1) ° and the phenylene ring at 37.6 (1)°. The amino group is hydrogen bond donor to the sulfonyl O atom of one molecule and to the pyrazolyl N atom of another molecule to result in the formation of a layer parallel to the bc plane.

### Experimental

1-Phenylbutan-1,3-dione (10 mmol) and 4-hydrazinobenzenesulfonamide hydrochloride (10 mmol) were heated in ethanol (50 ml) for 4 h; water was added to precipitate the product, which was collected and recrystallized from ethanol as light yellow crystals; m.p. 471–472 K.

### Refinement

Carbon bound H-atoms were placed in calculated positions [C–H 0.95 to 0.98 Å, *U*_iso_(H) 1.2–1.5*U*_eq_(C)] and were included in the refinement in the riding model approximation.

The amino H-atoms were located in a difference Fouier map and were freely refined.

### Crystal data

**Table:**

C_16_H_15_N_3_O_2_S	*F*(000) = 1312
*M**_r_* = 313.37	*D*_x_ = 1.320 Mg m^−^^3^
Monoclinic, *C*2/*c*	Cu *K*α radiation, λ = 1.54184 Å
Hall symbol: -C 2yc	Cell parameters from 2773 reflections
*a* = 28.2545 (8) Å	θ = 3.1–74.2°
*b* = 11.9135 (4) Å	µ = 1.91 mm^−^^1^
*c* = 9.3739 (3) Å	*T* = 100 K
β = 91.016 (3)°	Prism, light-yellow
*V* = 3154.85 (17) Å^3^	0.30 × 0.03 × 0.03 mm
*Z* = 8	

### Data collection

**Table:**

Agilent SuperNova Dual diffractometer with an Atlas detector	3137 independent reflections
Radiation source: SuperNova (Cu) X-ray Source	2689 reflections with *I* > 2σ(*I*)
Mirror	*R*_int_ = 0.035
Detector resolution: 10.4041 pixels mm^-1^	θ_max_ = 74.4°, θ_min_ = 3.1°
ω scans	*h* = −35→31
Absorption correction: multi-scan (*CrysAlis PRO*; Agilent, 2010)	*k* = −12→14
*T*_min_ = 0.598, *T*_max_ = 0.945	*l* = −11→9
6579 measured reflections	

### Refinement

**Table:**

Refinement on *F*^2^	Primary atom site location: structure-invariant direct methods
Least-squares matrix: full	Secondary atom site location: difference Fourier map
*R*[*F*^2^ > 2σ(*F*^2^)] = 0.041	Hydrogen site location: inferred from neighbouring sites
*wR*(*F*^2^) = 0.115	H atoms treated by a mixture of independent and constrained refinement
*S* = 1.03	*w* = 1/[σ^2^(*F*_o_^2^) + (0.0629*P*)^2^ + 1.4812*P*] where *P* = (*F*_o_^2^ + 2*F*_c_^2^)/3
3137 reflections	(Δ/σ)_max_ = 0.001
208 parameters	Δρ_max_ = 0.33 e Å^−^^3^
0 restraints	Δρ_min_ = −0.51 e Å^−^^3^

### Fractional atomic coordinates and isotropic or equivalent isotropic displacement parameters (Å^2^)

**Table:**

	*x*	*y*	*z*	*U*_iso_*/*U*_eq_	
S1	0.690362 (14)	0.48353 (4)	0.65383 (4)	0.01752 (14)	
O1	0.67646 (4)	0.41033 (11)	0.76803 (13)	0.0220 (3)	
O2	0.69504 (5)	0.60135 (11)	0.68142 (14)	0.0252 (3)	
N1	0.65210 (5)	0.46753 (14)	0.52785 (16)	0.0191 (3)	
H1	0.6414 (8)	0.395 (2)	0.516 (2)	0.026 (6)*	
H2	0.6583 (8)	0.5054 (18)	0.452 (2)	0.017 (5)*	
N2	0.88441 (5)	0.35478 (13)	0.48169 (15)	0.0189 (3)	
N3	0.89694 (5)	0.24415 (13)	0.47817 (17)	0.0217 (3)	
C1	0.96762 (7)	0.13366 (18)	0.4272 (3)	0.0341 (5)	
H1A	0.9686	0.0956	0.5199	0.051*	
H1B	1.0000	0.1470	0.3954	0.051*	
H1C	0.9508	0.0864	0.3573	0.051*	
C2	0.94240 (6)	0.24326 (17)	0.4411 (2)	0.0234 (4)	
C3	0.95933 (6)	0.35272 (17)	0.42301 (19)	0.0225 (4)	
H3	0.9904	0.3741	0.3974	0.027*	
C4	0.92187 (6)	0.42307 (16)	0.44972 (17)	0.0193 (4)	
C5	0.91994 (6)	0.54696 (16)	0.44973 (18)	0.0192 (4)	
C6	0.89996 (6)	0.60729 (16)	0.5616 (2)	0.0229 (4)	
H6	0.8878	0.5683	0.6413	0.028*	
C7	0.89779 (7)	0.72353 (17)	0.5573 (2)	0.0284 (4)	
H7	0.8840	0.7639	0.6335	0.034*	
C8	0.91585 (7)	0.78079 (17)	0.4409 (2)	0.0307 (5)	
H8	0.9138	0.8603	0.4365	0.037*	
C9	0.93685 (7)	0.72183 (18)	0.3313 (2)	0.0292 (4)	
H9	0.9499	0.7612	0.2531	0.035*	
C10	0.93889 (6)	0.60550 (17)	0.3355 (2)	0.0235 (4)	
H10	0.9533	0.5656	0.2600	0.028*	
C11	0.83692 (6)	0.38175 (15)	0.51674 (18)	0.0188 (4)	
C12	0.81362 (6)	0.47000 (16)	0.44808 (19)	0.0214 (4)	
H12	0.8285	0.5097	0.3733	0.026*	
C13	0.76862 (6)	0.49917 (16)	0.48990 (19)	0.0213 (4)	
H13	0.7529	0.5611	0.4465	0.026*	
C14	0.74629 (6)	0.43750 (15)	0.59607 (18)	0.0183 (4)	
C15	0.76849 (6)	0.34505 (15)	0.65805 (18)	0.0193 (4)	
H15	0.7523	0.3004	0.7256	0.023*	
C16	0.81447 (6)	0.31858 (15)	0.62039 (19)	0.0194 (4)	
H16	0.8305	0.2576	0.6652	0.023*	

### Atomic displacement parameters (Å^2^)

**Table:**

	*U*^11^	*U*^22^	*U*^33^	*U*^12^	*U*^13^	*U*^23^
S1	0.0180 (2)	0.0196 (2)	0.0150 (2)	−0.00041 (15)	0.00220 (16)	−0.00277 (15)
O1	0.0219 (6)	0.0280 (7)	0.0161 (6)	0.0002 (5)	0.0038 (5)	−0.0002 (5)
O2	0.0254 (7)	0.0217 (7)	0.0287 (7)	−0.0010 (5)	0.0032 (5)	−0.0067 (5)
N1	0.0192 (7)	0.0221 (8)	0.0159 (7)	−0.0009 (6)	0.0013 (6)	0.0001 (6)
N2	0.0169 (7)	0.0212 (8)	0.0187 (7)	0.0003 (6)	0.0012 (6)	−0.0010 (6)
N3	0.0201 (7)	0.0195 (8)	0.0256 (8)	0.0009 (6)	0.0010 (6)	−0.0027 (6)
C1	0.0271 (10)	0.0280 (11)	0.0474 (13)	0.0041 (8)	0.0067 (9)	−0.0085 (9)
C2	0.0198 (9)	0.0270 (10)	0.0234 (9)	0.0012 (7)	0.0000 (7)	−0.0044 (7)
C3	0.0179 (8)	0.0283 (10)	0.0212 (9)	−0.0012 (7)	0.0017 (7)	−0.0037 (7)
C4	0.0180 (8)	0.0249 (9)	0.0149 (8)	−0.0034 (7)	0.0001 (6)	−0.0002 (7)
C5	0.0148 (8)	0.0236 (9)	0.0190 (9)	−0.0015 (7)	−0.0018 (6)	0.0007 (7)
C6	0.0207 (9)	0.0264 (10)	0.0218 (9)	−0.0014 (7)	0.0010 (7)	0.0004 (7)
C7	0.0248 (9)	0.0269 (10)	0.0335 (11)	0.0031 (8)	−0.0015 (8)	−0.0050 (8)
C8	0.0288 (10)	0.0219 (10)	0.0410 (12)	0.0007 (8)	−0.0067 (8)	0.0047 (9)
C9	0.0278 (10)	0.0302 (10)	0.0293 (10)	−0.0067 (8)	−0.0037 (8)	0.0098 (8)
C10	0.0206 (9)	0.0289 (10)	0.0211 (9)	−0.0038 (7)	0.0004 (7)	0.0009 (7)
C11	0.0161 (8)	0.0223 (9)	0.0179 (8)	−0.0009 (7)	−0.0001 (6)	−0.0023 (7)
C12	0.0189 (9)	0.0279 (10)	0.0175 (9)	−0.0022 (7)	0.0014 (7)	0.0054 (7)
C13	0.0186 (8)	0.0254 (9)	0.0199 (9)	−0.0003 (7)	−0.0003 (7)	0.0036 (7)
C14	0.0171 (8)	0.0210 (9)	0.0169 (8)	−0.0025 (7)	0.0005 (6)	−0.0031 (7)
C15	0.0220 (9)	0.0189 (9)	0.0171 (8)	−0.0028 (7)	0.0028 (7)	−0.0016 (7)
C16	0.0209 (9)	0.0192 (9)	0.0182 (8)	0.0003 (7)	0.0010 (6)	−0.0004 (7)

### Geometric parameters (Å, °)

**Table:**

S1—O2	1.4330 (13)	C6—C7	1.387 (3)
S1—O1	1.4407 (13)	C6—H6	0.9500
S1—N1	1.5983 (15)	C7—C8	1.391 (3)
S1—C14	1.7666 (18)	C7—H7	0.9500
N1—H1	0.92 (2)	C8—C9	1.387 (3)
N1—H2	0.86 (2)	C8—H8	0.9500
N2—N3	1.365 (2)	C9—C10	1.388 (3)
N2—C4	1.372 (2)	C9—H9	0.9500
N2—C11	1.424 (2)	C10—H10	0.9500
N3—C2	1.337 (2)	C11—C16	1.391 (2)
C1—C2	1.494 (3)	C11—C12	1.392 (3)
C1—H1A	0.9800	C12—C13	1.382 (3)
C1—H1B	0.9800	C12—H12	0.9500
C1—H1C	0.9800	C13—C14	1.397 (2)
C2—C3	1.400 (3)	C13—H13	0.9500
C3—C4	1.376 (3)	C14—C15	1.390 (3)
C3—H3	0.9500	C15—C16	1.389 (2)
C4—C5	1.477 (3)	C15—H15	0.9500
C5—C10	1.393 (3)	C16—H16	0.9500
C5—C6	1.398 (3)		
			
O2—S1—O1	118.97 (8)	C7—C6—H6	119.7
O2—S1—N1	108.01 (8)	C5—C6—H6	119.7
O1—S1—N1	106.68 (8)	C6—C7—C8	119.71 (19)
O2—S1—C14	106.21 (8)	C6—C7—H7	120.1
O1—S1—C14	107.27 (8)	C8—C7—H7	120.1
N1—S1—C14	109.49 (8)	C9—C8—C7	120.03 (19)
S1—N1—H1	114.6 (14)	C9—C8—H8	120.0
S1—N1—H2	113.6 (14)	C7—C8—H8	120.0
H1—N1—H2	117.8 (19)	C8—C9—C10	120.20 (18)
N3—N2—C4	111.46 (14)	C8—C9—H9	119.9
N3—N2—C11	117.98 (14)	C10—C9—H9	119.9
C4—N2—C11	130.56 (16)	C9—C10—C5	120.36 (18)
C2—N3—N2	105.35 (15)	C9—C10—H10	119.8
C2—C1—H1A	109.5	C5—C10—H10	119.8
C2—C1—H1B	109.5	C16—C11—C12	120.91 (16)
H1A—C1—H1B	109.5	C16—C11—N2	118.85 (16)
C2—C1—H1C	109.5	C12—C11—N2	120.24 (16)
H1A—C1—H1C	109.5	C13—C12—C11	119.26 (16)
H1B—C1—H1C	109.5	C13—C12—H12	120.4
N3—C2—C3	110.85 (16)	C11—C12—H12	120.4
N3—C2—C1	119.44 (18)	C12—C13—C14	119.97 (17)
C3—C2—C1	129.70 (17)	C12—C13—H13	120.0
C4—C3—C2	106.24 (16)	C14—C13—H13	120.0
C4—C3—H3	126.9	C15—C14—C13	120.57 (16)
C2—C3—H3	126.9	C15—C14—S1	121.16 (13)
N2—C4—C3	106.09 (16)	C13—C14—S1	118.22 (14)
N2—C4—C5	124.32 (16)	C16—C15—C14	119.42 (16)
C3—C4—C5	129.57 (16)	C16—C15—H15	120.3
C10—C5—C6	119.01 (18)	C14—C15—H15	120.3
C10—C5—C4	119.05 (16)	C15—C16—C11	119.67 (16)
C6—C5—C4	121.94 (16)	C15—C16—H16	120.2
C7—C6—C5	120.64 (18)	C11—C16—H16	120.2
			
C4—N2—N3—C2	−1.13 (19)	C6—C5—C10—C9	−1.6 (3)
C11—N2—N3—C2	179.13 (15)	C4—C5—C10—C9	178.87 (16)
N2—N3—C2—C3	1.0 (2)	N3—N2—C11—C16	37.9 (2)
N2—N3—C2—C1	179.95 (17)	C4—N2—C11—C16	−141.76 (18)
N3—C2—C3—C4	−0.5 (2)	N3—N2—C11—C12	−142.14 (17)
C1—C2—C3—C4	−179.3 (2)	C4—N2—C11—C12	38.2 (3)
N3—N2—C4—C3	0.86 (19)	C16—C11—C12—C13	3.9 (3)
C11—N2—C4—C3	−179.44 (16)	N2—C11—C12—C13	−176.02 (16)
N3—N2—C4—C5	−177.79 (15)	C11—C12—C13—C14	−2.5 (3)
C11—N2—C4—C5	1.9 (3)	C12—C13—C14—C15	−1.5 (3)
C2—C3—C4—N2	−0.24 (19)	C12—C13—C14—S1	176.01 (14)
C2—C3—C4—C5	178.31 (17)	O2—S1—C14—C15	128.99 (15)
N2—C4—C5—C10	−133.78 (18)	O1—S1—C14—C15	0.77 (17)
C3—C4—C5—C10	47.9 (3)	N1—S1—C14—C15	−114.63 (15)
N2—C4—C5—C6	46.7 (2)	O2—S1—C14—C13	−48.55 (16)
C3—C4—C5—C6	−131.6 (2)	O1—S1—C14—C13	−176.76 (14)
C10—C5—C6—C7	1.8 (3)	N1—S1—C14—C13	67.84 (16)
C4—C5—C6—C7	−178.70 (16)	C13—C14—C15—C16	4.2 (3)
C5—C6—C7—C8	−0.3 (3)	S1—C14—C15—C16	−173.24 (13)
C6—C7—C8—C9	−1.4 (3)	C14—C15—C16—C11	−2.8 (3)
C7—C8—C9—C10	1.6 (3)	C12—C11—C16—C15	−1.2 (3)
C8—C9—C10—C5	−0.1 (3)	N2—C11—C16—C15	178.71 (15)

### Hydrogen-bond geometry (Å, °)

**Table:**

*D*—H···*A*	*D*—H	H···*A*	*D*···*A*	*D*—H···*A*
N1—H1···N3^i^	0.92 (2)	1.98 (2)	2.878 (2)	164 (2)
N1—H2···O1^ii^	0.86 (2)	2.07 (2)	2.930 (2)	177 (2)

Symmetry codes: (i) −*x*+3/2, −*y*+1/2, −*z*+1; (ii) *x*, −*y*+1, *z*−1/2.
